# Association of Myelofibrosis Phenotypes with Clinical Manifestations, Molecular Profiles, and Treatments

**DOI:** 10.3390/cancers15133331

**Published:** 2023-06-24

**Authors:** Helen T. Chifotides, Srdan Verstovsek, Prithviraj Bose

**Affiliations:** Department of Leukemia, The University of Texas MD Anderson Cancer Center, Houston, TX 77030, USA; htchifotides@mdanderson.org (H.T.C.); srdanverstovsek@gmail.com (S.V.)

**Keywords:** anemia, cytopenic, high molecular risk mutations, momelotinib, myelodepletive, myelofibrosis, PMF, MPN, myeloproliferative neoplasm, pacritinib, phenotype, thrombocytopenia

## Abstract

**Simple Summary:**

Myelofibrosis is an aggressive bone marrow cancer whose clinical presentation can be extremely heterogenous. Two distinct phenotypes, myeloproliferative and myelodepletive or cytopenic, have increasingly been recognized in recent years. The two phenotypes represent the two ends of the disease spectrum and are characterized by opposing trends for a wide range of clinical variables (e.g., peripheral blood counts, spleen volume) and molecular profiles that result in significantly different prognoses and outcomes. The myeloproliferative phenotype is usually associated with normal/higher peripheral blood counts and larger spleen volume, higher mutant *JAK2* allele burden, fewer “non-driver mutations”, and superior overall survival. The myelodepletive phenotype is associated with progressive anemia and/or thrombocytopenia, modest splenomegaly, more high molecular risk mutations, lower mutant *JAK2* allele burden, and inferior outcomes. Management of myelofibrosis is largely dictated by clinical needs, including the degrees of splenomegaly, symptoms and cytopenias, as well as prognostic risk assessment. Ruxolitinib and fedratinib are more efficacious in the myeloproliferative phenotype, whereas momelotinib and pacrtitinib can address the unmet needs of the myelodepletive phenotype.

**Abstract:**

Myelofibrosis (MF) presents an array of clinical manifestations and molecular profiles. The two distinct phenotypes− myeloproliferative and myelodepletive or cytopenic− are situated at the two poles of the disease spectrum and are largely defined by different degrees of cytopenias, splenomegaly, and distinct molecular profiles. The myeloproliferative phenotype is characterized by normal/higher peripheral blood counts or mildly decreased hemoglobin, progressive splenomegaly, and constitutional symptoms. The myeloproliferative phenotype is typically associated with secondary MF, higher *JAK2* V617F burden, fewer mutations, and superior overall survival (OS). The myelodepletive phenotype is usually associated with primary MF, ≥2 cytopenias, modest splenomegaly, lower *JAK2* V617F burden, higher fibrosis, greater genomic complexity, and inferior OS. Cytopenias are associated with mutations in epigenetic regulators/splicing factors, clonal evolution, disease progression, and shorter OS. Clinical variables, in conjunction with the molecular profiles, inform integrated prognostication and disease management. Ruxolitinib/fedratinib and pacritinib/momelotinib may be more suitable to treat patients with the myeloproliferative and myelodepletive phenotypes, respectively. Appreciation of MF heterogeneity and two distinct phenotypes, the different clinical manifestations and molecular profiles associated with each phenotype alongside the growing treatment expertise, the development of non-myelosuppressive JAK inhibitors, and integrated prognostication are leading to a new era in patient management. Physicians can increasingly tailor personalized treatments that will address the unique unmet needs of MF patients, including those presenting with the myelodepletive phenotype, to elicit optimal outcomes and extended OS across the disease spectrum.

## 1. Introduction

**Figure 1:**
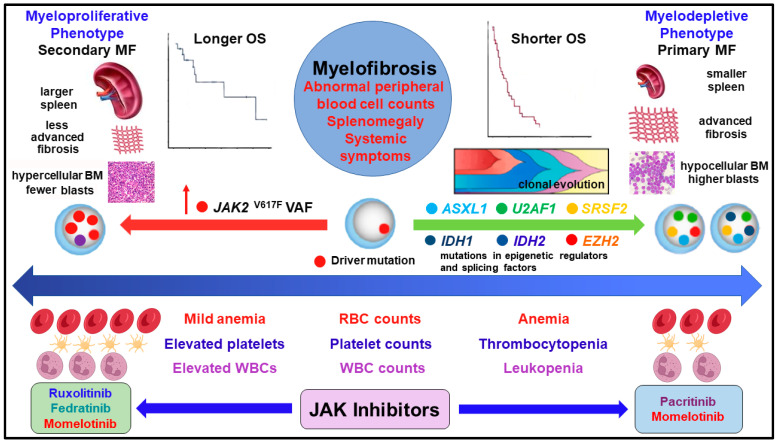
MF phenotypes (myeloproliferative and myelodepletive), clinical and molecular characteristics, and suggested treatment options with JAK inhibitors. Abbreviations. BM: bone marrow; OS: overall survival; RBC: red blood cell; VAF: variant allele frequency; WBC: white blood cell.

Myelofibrosis (MF) is the most aggressive among the classic myeloproliferative neoplasms (MPN), which are diseases of the blood/bone marrow [1] characterized by extensive heterogeneity in clinical manifestations and molecular markers. Two distinct clinical phenotypes (myeloproliferative and myelodepletive or cytopenic) have been recognized in MF and are determined by several clinical features (primarily peripheral blood counts, splenomegaly and disease trajectory) and molecular profiles; both play pivotal roles in prognosis and outcomes [2,3]. The underlying biology of the two phenotypes is different, and the relationship between the molecular profiles and each phenotype is complex and multifactorial. The myelodepletive phenotype (Table 1, Figure 1) is typically associated with primary MF (PMF) [4] and cytopenias (anemia and/or thrombocytopenia or severe pancytopenia). Patients with myelodepletive MF often require red blood cell and/or platelet transfusion support [5] and present with circulating blasts and bone marrow fibrosis grade 2 or higher [2,3]. The myelodepletive phenotype behaves similarly to a state of bone marrow failure [2,3]. On the other hand, the myeloproliferative phenotype (Table 1; Figure 1) is usually associated with the evolution of polycythemia vera (PV) or essential thrombocythemia (ET) to secondary MF. In the myeloproliferative phenotype, there is an expansion of one or more myeloid lineages, evidenced by normal or high blood counts, such as leukocytosis and/or elevated platelet counts. Patients with the myeloproliferative phenotype have normal to mildly decreased hemoglobin (Hb), and the need for transfusion support is minimal in these patients; the bone marrow is hypercellular, and patients often exhibit large splenomegaly and constitutional symptoms [2]. In a retrospective study of 1099 patients with primary and secondary MF, more patients with PMF were transfusion-dependent and had thrombocytopenia, whereas more patients with post-PV MF had leukocytosis and constitutional symptoms [6]. Although cytopenias are associated more with the myelodepletive phenotype [2], they are inevitable in the majority of patients with advanced MF (primary and secondary).

In this article, we comprehensively review the clinical manifestations and molecular profiles associated with the two distinct phenotypes of MF, the impact of cytopenias, and driver and non-driver mutations on prognostication, and provide an overview of the approved/emerging JAK inhibitors in the treatment of each phenotype. The two non-myelosuppressive JAK inhibitors (pacritinib, momelotinib) are evolving into treatments of choice for the myelodepletive phenotype, which is associated with cytopenias and other high-risk features and, historically, poor outcomes. The armamentarium of approved JAK inhibitors and other regimens is expanding to target alternate biological pathways besides the Janus kinase/signal transducers and activators of transcription (JAK-STAT) signaling pathway, splenomegaly, and constitutional symptoms, which have conventionally been targeted in MF until now. The phenotype and types of cytopenia(s) will also inform the design of personalized treatments, leading to superior outcomes across the MF disease spectrum in the near future.

## 2. Driver and Non-Driver Mutations, Clonal Evolution, and Cooperating Mutations Associated with Disease Progression and Survival

The advent of next-generation sequencing (NGS) demonstrated that MF exhibits notable heterogeneity, clonal dynamics, and complexity at a molecular level besides a range of clinical manifestations. PMF and secondary MF are considered distinct entities with respect to their biology and molecular profiles. Constitutive activation of the JAK/STAT signaling pathway has a fundamental role in MF biology [7]. Approximately 60%, 25–30%, and 5–10% of PMF patients harbor *JAK2* V617F, *CALR* exon 9, and *MPL* (most commonly W515L/K) mutations, respectively [1,2,3,8]. *JAK2* mutations (V617F and exon 12) are nearly universal in patients with post-PV MF, whereas *JAK2* V617F and *CALR* exon 9 indels are detected in approximately 50% and 30% of patients with post-ET MF, respectively [9]. Typically, the myelodepletive and myeloproliferative phenotypes are associated with low and high *JAK2* V617F allele burden, respectively [2]. However, a correlation between the *JAK2* V617F allelic burden with advanced MF and cytopenias has not been universally found. For example, in a retrospective study of patients with secondary MF, the *JAK2* V617F status/allele burden did not influence the clinical phenotype and prognosis [10]. Coltro et al. performed a phenotypic and mutational investigation of 704 patients with PMF and secondary MF [11]; in this study, no correlations were found between the *JAK2* V617F status/allele burden and the myelodepletive phenotype in the PMF cohort [11].

Beyond the three oncogenic driver mutations (*JAK2* V617F, *MPL* W515L, and *CALR*) [12,13,14], cooperation with other non-driver mutations and/or other genomic aberrations is required for disease progression [14,15,16,17,18]. Clonal evolution of the principal clone can generate subclones by acquiring new mutations over time, or the transformation process can be biclonal (co-existing clones) from the outset [12,15,18,19,20,21]. The founding clone can be mutated*-JAK2* V617F, or *JAK2* V617F can be preceded by the acquisition of another mutation, for example, in an epigenetic regulator [12,15,22]. The order in which the mutations are acquired (driver mutation first, mutation in the epigenetic regulator second, or *vice versa*) plays an important role in clonal dynamics (competing clones) and proliferation and progression of the disease [15,21,22,23].

Non-driver mutations implicated in MF pathogenesis span genes involved in epigenetic regulation (*ASXL1*, *EZH2, IDH1*, *IDH2*, *TET2*) [22], mRNA splicing (*U2AF1*, *SRSF2, SF3B1*), transcriptional regulation (*TP53*, *NFE2*, *RUNX1*), and signaling (*NRAS*/*KRAS*, *CBL*) [15,18,24]. Mutations in epigenetic regulators (*ASXL1*, *EZH2, IDH1*, *IDH2)* and mRNA splicing factors (*SRSF2*, *SF3B1*) are the most common (*ASXL1* is the most frequent) [12,15,18,25] and prognostically informative. In a multinational study of 879 patients with PMF, Vannucchi et al. identified five high molecular risk (HMR) mutations (harbored by 25–30% of the patients with PMF) that affected leukemia-free survival (LFS) and OS (*ASXL1*, *EZH2, IDH1*, *IDH2*, *SRSF2*) [26]. In a preceding analysis of 63 specimens from patients with MPN in the blast phase, *ASXL1* mutations were detected in paired specimens during both chronic and blast phase MPN, thereby implicating *ASXL1* mutations in leukemic transformation, while *TET2* mutations were frequently acquired during leukemic transformation [27]. Paz et al. reported an association of high-risk mutations (*EZH2*, *IDH1/2*, *SRSF2*, *N/KRAS*, *U2AF1*, *CBL*) with leukemic transformation and inferior survival in a recent study of 497 patients with primary and secondary MF [28]. The authors also reported that *ASXL1* mutations were prognostically significant in leukemic transformation and survival only when *ASXL1* mutations were associated with *TP53* or other high-risk mutations (*EZH2*, *IDH1/2*, *SRSF2*, *N/KRAS*, *U2AF1*, *CBL*) [28]. In a recent study that included cohorts with PMF and secondary MF, Guglielmelli and Coltro et al. confirmed the strong negative prognostic impact of *ASXL1* mutations with or without co-occurring HMR mutations (*EZH2*, *IDH1/2*, *SRSF2*, *U2AF1*) in PMF but not in secondary MF [29]. This finding is in accordance with the results of Wang et al., who reported that the co-occurrence of the *ASXL1* mutation with low *JAK2* V617F variant allele frequency (VAF) is prognostically adverse, compared to the co-existence of mutated *ASXL1* with high *JAK2* V617F VAF in PMF patients [30]. In another study of 1306 patients who were monitored for 5 years after PMF diagnosis, mutated *ASXL1* (*p* = 0.01), *IDH1* (*p* = 0.02) and *SRSF2* (*p* = 0.001) had prognostic significance regarding disease progression [31]. *SRSF2* mutations are frequently detected in PMF patients, cluster with *IDH* mutations, and are associated with poor survival [32]. In a study of 520 patients, Loscocco et al. found a negative impact of *SF3B1* mutations on OS in patients with secondary MF (HR 3.2, *p* = 0.002) but not PMF (HR = 1.1, *p* = 0.8), whereas *SF3B1* mutations had no impact on leukemia-free survival in either cohort [33].

Several studies demonstrated that HMR mutations are typically acquired during the course of the disease and as MF progresses to the blast phase [12,15,16,18,20]. Mutations in epigenetic regulators frequently co-occur with driver mutations, are significantly enriched in MF compared to PV and ET and have been associated with myelofibrotic progression and leukemic transformation [22]. Mutations in epigenetic regulators, splicing factors and the RAS pathway had a strong association with the progression of chronic phase MPN to the accelerated and blast phases [17,19,20,34]. Cooperation of *IDH1/2* mutations with *JAK2* V617F in leukemic transformation was demonstrated in a study of 301 patients with PMF who progressed to the blast phase: concomitant mutations *IDH* and *JAK2* V617F resulted in a more pronounced effect on leukemia-free survival and OS (*p* < 0.0001, *p* = 0.0002, respectively) compared to the absence of *IDH* mutations [35]. The cooperation of *Idh2* with *Jak2* V617F mutations [36] and loss of *Ezh2* combined with *JAK2* V617F [37] resulted in the induction and progression of MPN in mouse models. In another study where paired MPN specimens from the chronic and blast phases were compared, mutations in *SRSF2*, *U2AF1*, and *IDH1/2* in combination with mutations in driver genes (*JAK2* V617F in the vast majority of cases) at the time of diagnosis were associated with rapid leukemic transformation; the mean time to disease progression was 1.5 years in patients harboring mutated *SRSF2* and *U2AF1* [38].

Two groups of investigators showed that loss of heterozygosity in *TP53* was associated with leukemic transformation, thereby tying wild-type *TP53* allele loss to clonal expansion [39,40]. Rampal et al. found that *TP53* mutations were more common in *JAK2* V617F-mutated specimens in the blast phase than in chronic phase MPN; the *TP53* allele burden was >50% at the time of leukemic transformation (vs. 7% in the chronic phase), and *TP53* nullizygosity potently cooperated with *JAK2* V617F to induce leukemic transformation in paired patient specimens [41]. Accordingly, a higher incidence of leukemic transformation was noted in a cohort of MF patients harboring *TP53* mutations concomitantly with *JAK2* V617F and *JAK2* “variants” [42]. Loss of the tumor suppressor *JARID2* preceded by the acquisition of *JAK2* V617F or *IDH2* R140Q mutations resulted in MPN acceleration or leukemogenesis in preclinical studies [43]. Notably, in hematopoietic stem and progenitor cells, loss of LKB1/*STK11* in cooperation with driver mutations promoted disease progression and leukemic transformation [44].

Several studies demonstrated the prognostic impact of the mutation number on disease progression. In an exome sequencing study that was conducted in 197 patients with MPN (34 had PMF), harboring ≥2 mutations increased the risk of leukemic transformation and reduced OS [40]. Another international study of 797 patients with PMF demonstrated the detrimental effect of harboring ≥2 HMR mutations (*ASXL1*, *EZH2*, *SRSF2*, or *IDH1/2*) with significantly shorter median leukemia-free survival as compared to one HMR mutation only and absence of HMR mutations (2.6, 7, and 12.3 years, respectively) [45]. In a group of patients with MPN in the accelerated/blast phase, acquisition of ≥4 mutations (detected in 46% of the patients) or mutated-*TP53* was associated with shorter survival [46]. An NGS study in 182 patients with PMF demonstrated an association between the increasing number of adverse mutations (*ASXL1*, *SRSF2*, *CBL*, *KIT*, *RUNX1*, *SH2B3*, and *CEBPA*) and median OS: none, 1 or 2, and 3 or more mutations corresponded to 8.5, 4, and 0.7 years, respectively (*p* < 0.001) [25]. MF patients who harbored *N/KRAS* mutations had a higher incidence of developing MPN in the blast phase at 3 years (*p* = 0.03) and a shorter 3-year OS (*p* < 0.001) [47]. MF patients harboring *RAS/CBL* mutations had a considerably higher 5-year cumulative incidence of leukemogenesis and inferior OS compared to the wild-type group [48]. PMF patients with *CALR*^−^*ASXL1*^+^ mutational status had considerably inferior OS (2.3 years) compared to *CALR*^+^ *ASXL1*^−^ patients who had the longest survival (9.6 years) [49]. In another PMF cohort, *JAK2* V617F-mutated patients had a higher incidence of co-occurring splicing mutations (*U2AF1* Q157, *SRSF2*) and were more likely to harbor ≥2 HMR mutations compared to *CALR*-mutated patients, thereby reinforcing the favorable prognostic impact of *CALR* mutations [50].

## 3. Genes Associated with the Myeloproliferative and Myelodepletive Phenotypes

The type of driver and non-driver or cooperating mutations, the allele burden, the order in which mutations were acquired, and other genomic factors have an impact on the phenotype [15,17]. Anemia and leukopenia, which are characteristics of the myelodepletive phenotype, were associated with a low *JAK2* V617F allele burden (<25%) and inferior survival [2,3,51]. PMF patients with the myelodepletive phenotype were more likely to be “triple negative”, namely lacking the three driver mutations (*p* < 0.0001), and harbored *ASXL1* (*p* = 0.0074), *IDH1/2* (*p* = 0.064), *N/KRAS* (*p* = 0.0014), *U2AF1* (*p* < 0.0001), and *CUX1* (*p* = 0.0002) mutations more often [11]. “Triple-negative” PMF patients are often older, have lower Hb levels, and platelet and leukocyte counts, in accordance with the myelodepletive phenotype [52], and characteristically have low platelet counts [11,49].

The myelodepletive phenotype is typically associated with a low *JAK2* allele burden [2]. The mutation profile can be further complicated by the acquisition of ≥3 high-risk mutations in epigenetic regulators and/or mRNA splicing factors, which amplify cytopenias [12,23]. Harboring ≥3 non-driver mutations contributes myelodysplastic features to the phenotype and increases the severity of the disease and the risk of evolution to the blast phase [3,12]. Notably, the critical role of mutations in epigenetic regulators and splicing factors, such as *ASXL1* [53,54], *SRSF2* [55] and *U2AF1* [56], in impaired hematopoiesis and the development of myelodysplastic syndromes (MDS), was validated in preclinical and clinical [57] studies. *ASXL1*, *TET2*, *U2AF1*, *SRSF2* and *SF3B1* mutations are frequently detected in MDS/MPN-unclassifiable, further supporting the development of myelodysplastic features in the MF phenotype (with the ensuing cytopenias) when ≥3 non-driver mutations are acquired [58]. Loscocco et al. found lower Hb levels in *SF3B1*-mutated MF patients compared to the wild type cohort [33]. Accordingly, in a retrospective study that we conducted, we found that patients with PMF and secondary MF harboring the *SF3B1* mutation had anemia and a high transfusion burden [59], in line with the impaired erythroid differentiation and anemia noted in *SF3B1-*mutated patients with MDS. Consistent with the aforementioned studies, spliceosome *U2AF1* mutations have been associated with the myelodepletive phenotype [11], severe anemia and thrombocytopenia, and smaller spleen size [25,60,61]; in particular, mutation *U2AF1* Q157 was associated with thrombocytopenia, anemia, and significantly shorter survival [61]. In another study, *U2AF1* mutations were strongly associated with cytopenic PMF and secondary MF with ≥2 cytopenias [11]. Similarly, thrombocytopenia was associated with low *JAK2* V617F VAF (*p* < 0.01), presence of *U2AF1* Q157 (*p* < 0.01), and ≥3 non-driver mutations (*p* < 0.01); harboring *SRSF2* or *TP53* significantly shortened OS in a large cohort of MF patients (with platelet counts <100 × 10^9^/L) [62]. Guglielmelli and Coltro et al. recently analyzed two distinct cohorts with PMF and secondary MF and found that in PMF patients, *ASXL1* mutations were associated with lower Hb levels (1.5 g/dL, *p* < 0.0001), more than two-fold higher transfusion dependence (*p* < 0.0001), nearly two-fold lower platelet counts (*p* < 0.0001), higher peripheral blasts (*p*< 0.0001), higher leukocyte counts (*p* = 0.0083), bone marrow fibrosis grade ≥2 (*p* < 0.0001), constitutional symptoms (*p* = 0.0001), advanced age (*p* < 0.0001), and male sex (*p* = 0.0042) [29]. Our group also demonstrated that higher circulating/bone marrow blasts (>5%) were associated with Hb levels <10 g/dL (*p* < 0.001), platelet counts <100 × 10^9^/L (*p* = 0.001), white blood cell counts >25 × 10^9^/L (*p* < 0.001), and reticulin fibrosis grade ≥2 (*p* = 0.03) [63]. Furthermore, *ASXL1* (47.7%) and *SRSF2* (14%) mutations were more common in PMF compared to secondary MF (27.1% and 3.4%), thereby correlating mutated *ASXL1* and *SRSF2* with the myelodepletive phenotype [64]. PMF patients harboring *EZH2* mutations had significantly higher leukocyte and blast cell counts and shorter OS compared to the wild type (*p* < 0.001) [65]. Accordingly, mutant-*ASXL1* patients with MF and an *Asxl1*^−/−^ *Jak2*^VF^ (*Asxl1* deletion/*Jak2* V617F) mouse model demonstrated accelerated bone marrow fibrosis compared to the wild-type *ASXL1* cohort and the *Jak2*^VF^ littermate, respectively [66]. Furthermore, a significant association was found between *ASXL1* mutations and fibrosis as well as disease progression in another recent study of 258 patients with PMF [67]. Notably, extremely high hazard ratios were reported for mutations in the nuclear factor erythroid-2 (*NFE2*) gene, a hematopoietic transcription factor, for leukemic transformation or progression to MDS and OS (10.3 and 8.24, respectively; *p* < 0.001); and the rates of hematological response to treatment were significantly lower (*p* = 0.026) [68].

The myeloproliferative phenotype is usually associated with a high *JAK2* V617F allele burden and the acquisition of fewer non-driver mutations compared to the myelodepletive phenotype [2,12]. In PMF, *JAK2* V617F has been associated with advanced age, higher Hb levels, lower platelet counts, and leukocytosis [12]. The median *JAK2* V617F allele burden in secondary MF (post-PV MF and post-ET MF: 92.6% and 62.6%, respectively) is significantly higher compared to PMF (48.8%), and nearly all post-PV MF patients harbor mutated *JAK2* [69,70]. A study that was conducted on 1099 patients who had primary or secondary MF demonstrated analogous results, namely that the median *JAK2* V617F allele burden in the post-PV MF cohort was 86% vs. 58% in the post-ET MF cohort vs. 47% in the PMF cohort [6]. The aforementioned findings corroborate another study in which a gradual increase in the *JAK2* V617F allele burden was the most powerful predictor of PV/ET progression to secondary MF during cytoreductive therapy (hazard ratio 10.8) [71]. Grinfeld et al. reported that *JAK2* V617F homozygosity or high *JAK2* allele burden was typically associated with PV, and myelofibrotic progression occurred more often in this cohort compared to the *JAK2*-heterozygous group (*p* = 0.007) [17]. Accordingly, Barosi et al. concluded that *JAK2* V617F homozygosity was independently associated with worse splenomegaly, higher white blood cell counts, aquagenic pruritus, and more frequent requirement for cytoreductive therapies in a study of 304 patients with PMF [72].

In a group of 227 patients with PMF, the cohort harboring *JAK2* V617F VAF ≥ 50% had a median OS of 80 months and higher Hb and white blood cell counts compared to the cohort with *JAK2* V617F VAF < 50%; the latter group had a median OS of 50 months (*p* = 0.01) [73]. In the same study, PMF patients with *JAK2* V617F VAF < 50% had a similar OS to “triple negative” patients (50 and 56 months, respectively) [73]. In accordance with the aforementioned studies, PMF patients who had *JAK2* V617F VAF in the range 1–20%, 21–55%, and 56–74% had a median survival of 20, 77, and 132 months, respectively (*p* = 0.0008) [74]. Accordingly, in another study of 370 patients with PMF, the cohort with *JAK2* V617F VAF <25% had shorter OS [65].

In a total of 617 patients with PMF from four centers, the cumulative incidence of developing anemia in *CALR*-mutated patients was lower compared to *JAK2*-mutated (*p* < 0.001), *MPL*-mutated (*p* = 0.004), and “triple-negative” (*p* < 0.001) patients; the likelihood that “triple-negative” patients would develop anemia was higher than in *CALR*-mutated (*p* < 0.001) or *JAK2*-mutated (*p* = 0.013) patients [52]. Regarding thrombocytopenia, *CALR*-mutant patients had the lowest cumulative incidence compared to *JAK2*- and *MPL*-mutant patients and “triple negative” (*p* = 0.001) patients. The *CALR*-mutated patients had a significantly lower cumulative incidence of developing leukocytosis compared to *JAK2* mutant patients (*p* = 0.004) [52]. *CALR* mutations in PMF were correlated with patients who were younger, less likely to be anemic and require transfusions, and had a higher platelet count and a lower incidence of leukocytosis [49,52]. Notably, Guglielmelli et al. recently found an association of high mutant *CALR* VAF (≥55%) with shorter anemia-free and leukocytosis-free survivals, and thereby, more aggressive disease compared to low *CALR* VAF (<55%) in a cohort of 620 patients who had PMF or secondary MF [75]. Interestingly, *CALR* type 2/type 2-like mutations were associated with higher median Hb levels, significantly higher platelet counts, and higher white blood cell counts compared to *CALR* type 1/type 1-like mutations in a small cohort of PMF patients [76]. *TP53* mutations were enriched (19%) in patients who had secondary MF and ≥2 cytopenias (*p* = 0.0024) [11].

## 4. Molecular Profiles of MF Phenotypes and Prognostication

In PMF, the initial prognostic stratification models have evolved from incorporating clinical parameters only (International Prognostic Scoring System: IPSS; Dynamic IPSS: DIPSS) to integrating clinical and molecular variables (Mutation-Enhanced International Prognostic Scoring System 70: MIPSS70, MIPSS70-plus, and MIPSS70-plus v.2.0) to stratification exclusively based on genetic data (molecular and karyotype) in the case of the Genetically Inspired Prognostic Scoring System (GIPSS) [8,77,78,79,80]. GIPSS and the mutation-enhanced models, which are applied in PMF patients, take into consideration HMR mutations in epigenetic regulators (*ASXL1*, *EZH2*, *IDH1, IDH2*) and mRNA splicing factors (*SRSF2, U2AF1* Q157), and the absence of type 1/type 1-like *CALR* as prognostically adverse features. MIPSS70 plus v.2.0 and GIPSS allocate one point for the presence of *U2AF1* Q157 and the absence of type 1/type 1-like *CALR*, and the three mutation-enhanced models (MIPSS70, MIPSS70-plus, and MIPSS70-plus v.2.0) also take into consideration the number of HMR mutations [8,77,78,79,80]. Two points are allocated for the absence of *CALR* mutations (all types) in the Myelofibrosis Secondary to PV and ET-Prognostic Model (MYSEC-PM) [8,77,78,79,80]. Recently, Mosquera-Orgueira et al. derived and validated the Artificial Intelligence Prognostic Scoring System for Myelofibrosis model, which was based on eight clinical variables at diagnosis, by analyzing data from 1617 MF patients who were included in the Spanish Myelofibrosis Registry [81]. Barbui et al. recently reported that PMF patients harboring *JAK2* V617 in combination with lower IPSS scores had a higher risk of thrombosis [82].

Determination of the mutational profile and timely identification of targetable mutations may inform a more refined risk stratification and personalized prognostication, thus, enabling personalized treatment and improving efficacy and OS [78]. For example, if the therapeutically targetable *IDH1/2* mutations are detected during the chronic or accelerated/blast phase MPN, treatment with IDH1 (ivosidenib, olutasidenib) or IDH2 (enasidenib) inhibitors alone or in combination with JAK inhibitors or hypomethylating agents may be considered [83,84,85]. Given the high prevalence of *IDH2* mutations in blast phase MPN [12,18] and the synergistic efficacy of JAK2 and IDH2 inhibitors in *IDH2*/*JAK2* mutated-MPN patient cells [36], a phase 2 clinical trial evaluating ruxolitinib in combination with enasidenib in *IDH2*-mutated patients with chronic MF (4–9% circulating blasts) and MF in the accelerated or blast phase is currently underway (NCT04281498) [86]. Notably, Grinfeld et al. designed a novel personalized prognostication model based on a study of 2035 MPN patients (MF, PV, ET) and integration of genomic data with clinical variables [17]. However, the molecular profiles of the patients may not always be available. In these cases, the IPSS, DIPSS, and DIPSS-plus (if cytogenetic data are available) can be applied [8,77,78,80]; disease progression can be assessed by monitoring splenomegaly, and the development of cytopenias, including transfusion requirements, over time. It is important to note that *JAK2* V617F can be detected in individuals with age-related clonal hematopoiesis or clonal hematopoiesis of indeterminate potential (ARCH/CHIP) who do not manifest an MF phenotype [18,87,88]. Background ARCH/CHIP may reflect the presence or persistence of mutations with low VAF [11,77]. ARCH mutations in three epigenetic regulators (*ASXL1*, *TET2*, *DNMT3A*) have been detected in healthy people aged 65 years or more, indicating that the molecular profile should be interpreted with caution in the absence of hematological/clinical abnormalities [89].

## 5. Prognostic Relevance of Cytopenias Encountered in the Myelodepletive Phenotype and Patient Outcomes

In MF patients, cytopenias can be disease-related, treatment-related, or due to a combination of both because ruxolitinib (JAK1/2 inhibitor) and fedratinib (JAK2 inhibitor) can exacerbate cytopenias [90,91,92,93,94,95]. Progression to cytopenic MF may indicate that the disease was preceded by a chronic, less aggressive phase. Conversely, presentation with cytopenic MF indicates an aggressive myelodepletive phenotype, which is associated with increasing cytopenias, the requirement for transfusions, bleeding manifestations, and poor survival [96]. Notably, the cytopenic phenotype was associated with inferior survival in cohorts with prefibrotic and overt PMF (*U2AF1* mutations were enriched in both) compared to the myeloproliferative phenotype; the incidence of the cytopenic phenotype was 49% vs. 23% in overt PMF and prefibrotic PMF, respectively (*p* < 0.0001) [97].

The profound impact of severe anemia in particular, and to a lesser extent thrombocytopenia, as high-risk factors for disease progression in MF is underscored by being featured in all the prognostic models for PMF (DIPSS-plus and MIPSS70 for platelet counts) [80]. Anemia is one of the cardinal features of MF, its pathogenesis is multifactorial and not fully understood, and it negatively affects prognosis [94,95,98]. Severe, transfusion-dependent anemia increases the risk of death in PMF patients by a factor of 1.5 compared to moderate anemia [99]. Red blood cell (RBC) transfusion dependence significantly decreased survival to 2.6 years in PMF patients who required transfusions at diagnosis compared to 8 years in patients who did not (*p* < 0.001) [100]. The importance of anemia as a risk factor in MF is evidenced by the fact that Hb < 10 g/dL is included in the IPSS, DIPSS/DIPSS-plus, and MIPSS70/MIPSS70-plus v.2.0 prognostic models; and sex- and severity-adjusted Hb levels are incorporated in the MIPSS70-plus v.2.0 [80]. Hb < 11 g/dL is included in MYSEC-PM (higher threshold because post-PV/ET MF patients tend to have milder cytopenias); moreover, besides Hb < 10 g/dL, transfusion-dependence is included in the DIPSS-plus model [80].

At initial diagnosis of PMF, about 40% of the patients are anemic (Hb < 10 g/dL), and nearly 25% require RBC transfusions; the prevalence of transfusion-dependence increased to nearly 50% one year after diagnosis [101]. In a retrospective study, transfusion dependence was higher in patients with PMF compared to those with post-PV MF and post-ET MF (29% vs. 17% vs. 20%, respectively); Hb < 10 g/dL remained a significant prognostic factor for inferior OS in PMF, post-PV MF, and post-ET MF in univariate and multivariate analyses [6]. Anemia and thrombocytopenia often coexist: in a study of thrombocytopenic patients with primary and secondary MF, patients with platelet counts below 50 × 10^9^/L had the highest rate of RBC transfusion dependence (69%, 24%, and 40% in PMF, post-PV MF, and post-ET MF, respectively); the corresponding rates for patients with platelets in the range 50–100 × 10^9^/L were 37%, 41%, and 43%, respectively [102].

Thrombocytopenia arises from multiple factors, including displacement of medullary thrombopoietic tissue by fibrosis, JAK-inhibitor induced myelosuppression, and genetic factors (*U2AF1* mutations and complex/high-risk cytogenetics) [103]. In a retrospective study of 1269 patients with primary and secondary MF, OS was stratified according to platelet count range; OS decreased dramatically for patients with platelet counts below 50 × 10^9^/L. For platelet counts in the range < 50 × 10^9^/L, 50–100 × 10^9^/L, and >100 × 10^9^/L, OS was 15 months, 44 months, and 55 months, respectively [102]. Progression of PMF increased the rate of thrombocytopenia: in a study of 1000 patients with PMF, the prevalence of thrombocytopenia increased from 18% to 28% between patients referred at the time of initial diagnosis and those referred within 1 year of diagnosis [101]. Accordingly, in another study, severe thrombocytopenia was proposed as an accelerated phase-defining feature in PMF with short median OS (12 months) [104].

Platelet counts <100 × 10^9^/L were allocated one point in the DIPSS-plus and two points in the MIPSS70 prognostic models, respectively, for primary MF, whereas platelet counts <150 × 10^9^/L were allocated one point in the MYSEC-PM for secondary MF (higher threshold as post-PV/ET MF patients tend to have milder cytopenias) [80]. Thrombocytopenic PMF patients appear to have a worse prognosis compared to patients with secondary MF and thrombocytopenia, and the prevalence of thrombocytopenia is higher in PMF. In a retrospective study of 1109 MF patients, the cohort with platelet counts >100 × 10^9^/L had significantly superior survival compared to the cohorts with platelet counts in the range 50–100 × 10^9^/L and <50 × 10^9^/L (88.8 vs. 33.8 vs. 14.7 months, respectively); the cohort with the worst survival primarily comprised patients with PMF and high-grade bone marrow fibrosis [102]. In a study that included 1269 patients with PMF (877), post-PV MF (212), and post-ET MF (180), the PMF cohort had the worst prognosis and inferior OS compared to post-PV MF and post-ET patients for platelet counts >100 × 10^9^/L (50 months vs. 64 vs. 79 months, *p* = 0.001); however, platelet counts <50 × 10^9^/L did not appear to affect OS, in post-PV MF. In the same study, patients with post-ET MF and platelets below 50 × 10^9^/L had the shortest survival, worse than PMF and post-PV MF (median 6 vs. 15 vs. 20 months, respectively; *p* = 0.003) [102].

## 6. Phenotypes, Molecular Profiles, and Differentiated Efficacy of Treatments in MF

In light of the increasing use of NGS in clinical practice and the prognostic impact of several mutations being well established, the molecular profile of the patient at diagnosis and follow-up can inform treatment choices and identify patients at high risk of disease progression. Single-cell analysis conducted by Mylonas et al. in specimens of 15 patients with PMF or secondary MF at several time points during treatment with JAK inhibitors demonstrated clonal evolution characterized by the acquisition of new mutations and copy number alterations over time (mean follow-up 3.9 years) [20]. In a phase 1/2 study, NGS analysis of specimens from 95 MF patients who were treated with ruxolitinib demonstrated that patients with 1, 2, or more mutations in genes *ASXL1*, *EZH2*, and *IDH1/2* (for sole *ASXL1 p* < 0.001; for sole *EZH2 p* = 0.002) were considerably less likely to exhibit a spleen response to ruxolitinib and had shorter survival and time to treatment discontinuation [105]. Patients with more than three mutations of any type had nine-fold lower odds of having a spleen response to ruxolitinib (compared to patients with ≤2 mutations) and considerably shorter time to treatment discontinuation [105]. Association of *ASXL1* (HR = 1.86; *p* = 0.03) and *EZH2* (HR = 2.94; *p* = 0.009) mutations and an HMR mutation profile with shorter time to treatment failure with JAK1/2-inhibitors (ruxolitinib, momelotinib) and the independent associations of *ASXL1* or *EZH2* mutations with inferior OS were corroborated by multivariate analysis in another study [106].

Another group of MF patients who were monitored for 30 months had lower spleen and symptom responses to JAK inhibitors at 6 months and throughout treatment when harboring mutations in the RAS/MAPK pathway genes (*NRAS*, *KRAS*, *CBL*) compared to wild-type patients who had 59% response rate at 6 months [48]. However, Santos et al. reported a 2-year non-significantly longer OS in MF patients harboring RAS pathway mutations treated with ruxolitinib compared to patients who did not receive ruxolitinib [47].

In a study that included 46 patients with PMF or secondary MF receiving ruxolitinib, the spleen response rate did not depend on the type of driver mutation and high molecular risk profile at baseline; however, a high molecular risk profile and harboring *ASXL1* as the sole additional mutation predicted loss of spleen response in 3 years [107]. Furthermore, a decrease in the *JAK2* V617F allele burden by ≥20% was associated with spleen response duration, whereas an increase in non-driver mutation allele burden and clonal evolution correlated with loss of spleen response and treatment discontinuation [107].

In a retrospective study of MF patients, acquisition of the *ASXL1* mutation while being treated with ruxolitinib was associated with high white blood cell counts and mild thrombocytopenia at discontinuation of ruxolitinib [108]. *ASXL1* was the most frequently acquired mutation during clonal evolution; patients who experienced clonal evolution while on ruxolitinib had inferior survival (OS was 6 months) compared to those who did not (16 months) [108]. Spleen responses to ruxolitinib treatment (after dose adjustment) were associated with *JAK2* V617F allele burden ≥50%; in particular, patients with *JAK2* V617F allele burden ≥50% had a 5.5-fold higher probability of spleen response as compared to patients with *JAK2* V617F allele burden <50% or any other driver mutation [109]. In another recent study, Palandri et al. assessed the efficacy of ruxolitinib in 801 MF patients presenting with the myeloproliferative or myelodepletive phenotypes; the investigators noted significantly lower spleen responses in patients who had anemia and thrombocytopenia, considerably higher rates of ruxolitinib discontinuation in patients with ≥2 cytopenias (*p* = 0.03), and shorter median OS in patients with myelodepletive compared to the myeloproliferative phenotype (4.5 vs. 5.7 years; *p* = 0.03) [110]. Another large study, conducted by Palandri et al., confirmed that ruxolitinib was administered at lower doses and had lower clinical efficacy (in terms of spleen and constitutional symptom responses) at 6 months, and the survival was shorter in 407 patients presenting with the cytopenic versus the myeloproliferative phenotype (PMF or secondary MF), but the cumulative risk of progression to the blast phase was similar [111]. The cumulative incidence of ruxolitinib discontinuation was 57% and 38% at 5 years in patients presenting with the cytopenic and the myeloproliferative phenotype, respectively (*p* < 0.001); and more patients presenting with the cytopenic phenotype in both settings (PMF and secondary MF) discontinued ruxolitinib [111]. Notwithstanding that patients with the myelodepletive phenotype may have limited responses to ruxolitinib, MF patients who had baseline platelet counts ranging from 50 to <100 × 10^9^/L and were treated with lower starting doses of ruxolitinib (≤10 mg bid) experienced meaningful reductions in spleen size and improvements in constitutional symptoms in the EXPAND (NCT01317875) [112] and JUMP (NCT01493414) [113] studies (Table 2). The pooled analysis of fedratinib’s efficacy at 400 mg daily in moderately thrombocytopenic (50 to <100 × 10^9^/L) MF patients who participated in the JAKARTA and JAKARTA2 trials was recently reported [114]; similarly to ruxolitinib, fedratinib may also exacerbate thrombocytopenia, but may be dosed at 400 mg daily (starting dose) in all patients with baseline platelets of ≥50 × 10^9^/L [90,91,93,114].

The dismal median OS noted in patients who discontinued ruxolitinib due to disease progression (e.g., cytopenias) [90,91,92,115], correlation of ruxolitinib dose with spleen response [91], and inclusion of ruxolitinib dose <20 mg bid (at baseline, month 3, and month 6) as an adverse prognostic risk factor in the RR6 model [116] underscore the critical need for non-myelosuppressive JAK inhibitors and other novel treatments in cytopenic patients with MF [87,91,92,117,118,119,120,121]. Pacritinib and momelotinib are non-myelosuppressive JAK inhibitors that have been approved or are in advanced clinical development and have demonstrated significant clinical benefits in MF patients manifesting features of the myelodepletive phenotype (cytopenias). Both momelotinib [122,123] and pacritinib [124] are potent inhibitors of activin A receptor, type 1, or activin receptor-like kinase-2 (ACVR1/ALK2); inhibiting aberrant activation of ACVR1/ALK2 suppresses the expression of hepcidin (master iron regulator) by hepatocytes, thereby restoring iron homeostasis and erythropoiesis, which leads to notable anemia benefits in MF patients [123].

Pacritinib also is a selective inhibitor of JAK2 (spares JAK1) and interleukin-1 receptor-associated kinase 1 (IRAK1) [3,125,126] that received accelerated regulatory approval as a treatment for patients with intermediate or high-risk MF (primary or secondary) and platelets below 50 × 10^9^/L based on the results of the phase 3 PERSIST-2 trial (the study enrolled patients with platelet counts ≤100 × 10^9^/L; Table 2) [127]. The dose-finding phase 2 PAC203 trial of pacritinib included MF patients with severe thrombocytopenia (platelet counts <50 × 10^9^/L) [128]. In the phase 3 PERSIST-1 trial [129], which evaluated pacritinib vs. best available therapy (BAT, excluding ruxolitinib) in JAK inhibitor-naïve patients, a large proportion of the patients had clinical characteristics reflecting the myelodepletive phenotype and *JAK2* V617F VAF ≤50%: 80.9% had PMF, 45.2% had platelet counts below 100 × 10^9^/L, 43.5% had Hb < 10 g/dL, 20.9% were transfusion dependent, and 18.3% had hypocellular bone marrow vs. 37.4%, 20.7%, 29.8%, 9.2%, and 6.2%, respectively, of those whose *JAK2* V617F VAF was >50% [130]. A significant correlation between the percentage spleen volume reduction and decrease in *JAK2* V617F VAF at week 24 was noted in patients treated with pacritinib (*p* = 0.003); the OS was longer in pacritinib-treated patients who had a greater decrease in *JAK2* V617F VAF vs. patients with minimal decrease in *JAK2* VAF [130]. A retrospective analysis of the data from the PERSIST-1 and PERSIST-2 trials demonstrated that the percentage of patients with VAF ≤50% who were treated with BAT (ruxolitinib was included as BAT in PERSIST-2 only) and had spleen volume reduction ≥35% (SVR35) was significantly lower compared to patients treated with pacritinib (*JAK2* > 0–25%: BAT 0%, pacritinib 21%, *p* < 0.001; *JAK2* > 25–50%: BAT 0%, pacritinib: 15%, *p* = 0.020); conversely, in the cohort with *JAK2* > 50–75%, the differences in spleen responses between BAT and pacritinib were not statistically significant (*p* = 0.033) [4,131]. Furthermore, 23% of the *JAK2* wild-type patients who were treated with pacritinib achieved SVR35 versus 0% in the BAT group [4]. Notably, the average *JAK2* V617F VAF for patients enrolled in the PERSIST-1 and PERSIST-2 trials was 47% versus 84% in the COMFORT studies (evaluating ruxolitinib), from which patients with moderate and severe thrombocytopenia were excluded [4]. A retrospective analysis of pacritinib’s efficacy in severely thrombocytopenic (platelet counts <50 × 10^9^/L) patients with MF who participated in the PERSIST-1 and PERSIST-2 trials demonstrated the superiority of pacritinib compared to BAT in terms of SVR35 (23% vs. 2%, *p* = 0.0007) and ≥50% improvement in total symptom score (TSS50; 25% vs. 8%, *p* = 0.044) [132]. At present, pacritinib is being further evaluated in comparison to “physician’s choice” in patients with advanced MF and severe thrombocytopenia in the phase 3 PACIFICA trial (NCT03165734; Table 2) [133]. Retrospective analysis of the data acquired from the PERSIST-2 trial demonstrated that among non-transfusion-independent patients (at baseline) with platelet counts <100×10^9^/L, 24% in the pacritinib Arm achieved transfusion independence vs. 5% treated with BAT (no transfusions over any 12-week period throughout the study with no Hb level <8 g/dL) by week 24 [124]. Pacritinib’s clinical efficacy in patients manifesting the myelodepletive phenotype with pronounced thrombocytopenia may be a result of inhibiting IRAK1 and the nuclear factor-κB pathway [3,126], whereas pacritinib’s anemia benefits are attributed to ACVR1/ALK2 inhibition [124].

Currently, momelotinib is in advanced clinical development as it is advantageously positioned to treat the three cardinal features of MF: anemia (including reduction or elimination of RBC transfusions), splenomegaly, and constitutional symptoms, owing to its inhibitory activity on ACVR1/ALK2 underlying its anemia benefits [123] and inhibition of JAK1/2 underlying spleen and symptom responses [134]. In the registrational, randomized phase 3 trial MOMENTUM (NCT04173494), momelotinib was evaluated versus danazol in JAK-inhibitor-exposed, anemic (Hb < 10 g/dL) and symptomatic (TSS ≥ 10) patients with intermediate- or high-risk MF (platelet counts ≥25 × 10^9^/L) [135]. In this study, the respective rates of RBC transfusion independence compared to baseline were 31% for momelotinib versus 20% for danazol, respectively [136]. Furthermore, 23% and 25% of the patients treated with momelotinib reached SVR35 and TSS50 versus 3% and 9% for danazol at week 24, respectively [136]. Sustained anemia benefits were recorded in the phase 3 SIMPLIFY-1 trial, wherein 66.5% of JAK-inhibitor naïve patients treated with momelotinib achieved or maintained transfusion independence versus 49.3% ruxolitinib-treated patients at week 24 [137]. Similarly, in the phase 3 SIMPLIFY-2 trial, momelotinib elicited superior anemia benefits compared to BAT (89% ruxolitinib) in second-line MF patients: 43% versus 21% reached or maintained transfusion-independence, respectively, at week 24 [138]. On the basis of the aforementioned clinical trials, momelotinib will likely receive regulatory approval in 2023 as a treatment for symptomatic and anemic patients with MF; momelotinib may become the preferred treatment for MF patients with anemia, splenomegaly, and constitutional symptoms, especially in the second line setting [134,139]. Notably, retrospective analyses demonstrated that momelotinib maintained its efficacy in thrombocytopenic patients in the two SIMPLIFY-1 and SIMPLIFY-2 trials [140] as well as in the MOMENTUM trial (Table 2) [136,141].

**Table 2:** Clinical trials that evaluated JAK inhibitors and enrolled thrombocytopenic patients with MF.

JAK Inhibitor	Clinical Trial NCT Number	Phase	Lowest Platelet Counts of Enrolled Patients	Reference
Ruxolitinib	EXPAND (NCT01317875)	1b	50 to <100 × 10^9^/L	[112]
Ruxolitinib	JUMP (NCT01493414)	3b	50 to <100 × 10^9^/L	[113]
Fedratinib	Pooled analysis of JAKARTA (NCT01437787) and JAKARTA2 (NCT01523171)	JAKARTA (phase 3) JAKARTA2 (phase 2)	50 to <100 × 10^9^/L	[114]
Pacritinib	PERSIST-1 (NCT01773187)	3	No lower limit	[129]
Pacritinib	PERSIST-2 (NCT02055781)	3	≤100 × 10^9^/L	[127]
Pacritinib	PAC203 (NCT04884191)	2	<50 × 10^9^/L	[128]
Pacritinib	PACIFICA (NCT03165734)	3	<50 × 10^9^/L	[133]
Momelotinib	SIMPLIFY-1 (NCT01969838)	3	≥50 × 10^9^/L	[140]
Momelotinib	SIMPLIFY-2 (NCT02101268)	3	No lower limit	[140]
Momelotinib	MOMENTUM (NCT04173494)	3	≥25 × 10^9^/L	[136,141]

Luspatercept (activin receptor-ligand trap enhancing late-stage erythropoiesis [142]) was evaluated in MF patients with anemia in a phase 2 trial (NCT03194542): in Cohort 3B (transfusion-dependent patients on a stable dose of ruxolitinib), RBC transfusion-independence was achieved in 27% and 36% of the patients, respectively, over any 12 consecutive weeks during the first 24 weeks and for ≥12 consecutive weeks when the entire treatment period was assessed; RBC transfusion burden decreased by ≥50% over at least 12 weeks in 46% of the patients [143]. At present, luspatercept is being evaluated in the registrational, phase 3 trial INDEPENDENCE (NCT04717414) in MF patients who are receiving a stable dose of a JAK inhibitor and require RBC transfusions [144].

In the phase 2/3 BOREAS clinical trial evaluating navtemadlin (a potent and selective human double minute 2 inhibitor that restores activity of p53 and malignant cell apoptosis) in wild-type *TP53* patients with relapsed/refractory MF (NCT03662126), the VAF of driver mutations decreased by ≥20% in 34% of the patients, and the reduction in VAF significantly correlated with spleen responses (at the recommended dose of 240 mg q.d. administered on days 1–7/28); patients who experienced reduction of ≥20% in driver mutation burden had better spleen responses compared to those who had VAF reduction *<* 20% after treatment with navtemadlin (32% vs. 5% respectively; *p* = 0.0072) [145]. Moreover, a complete VAF reduction (below the detection limit) in driver or HMR mutations was noted in 29% of the patients [145]. Overall, a correlation between biomarkers of disease burden and clinical benefits of navtemadlin was noted [145,146].

Combination regimens of novel agents with ruxolitinib have exhibited synergism and are currently being explored in clinical trials [117,118]. In Arm 3 of the MANIFEST trial (NCT02158858), which evaluated pelabresib (inhibitor of bromodomain and extra-terminal proteins) in combination with ruxolitinib in 84 JAK inhibitor-naïve patients, clinical benefits were observed regardless of molecular markers, including HMR mutations [147,148]. Furthermore, the mean Hb level improved by 1.3 g/dL in 36% of the patients, and *JAK2* V617F VAF decreased by >25% in 29.5% of the patients at week 24; an association was found between these benefits and SVR35 response (*p* = 0.018) [148]. A randomized registrational phase 3 trial (MANIFEST-2; NCT04603495), evaluating pelabresib or placebo in combination with ruxolitinib in JAK inhibitor-naïve MF patients, is currently underway [149]. In Cohort 1a of the phase 2 REFINE trial, “add-on” of navitoclax (BcL2/-xl inhibitor) to ruxolitinib in patients with a suboptimal response on ruxolitinib monotherapy (median prior ruxolitinib duration 20 months) elicited spleen responses regardless of HMR mutation status or the number of mutated genes at baseline; *JAK2* V617F and *CALR* VAF decreased by 20% or more in 23% of the patients; additionally, Hb increased by ≥2 g/dL in 64% of the patients [150]. In Cohort 3 of the REFINE trial, which evaluated navitoclax in combination with ruxolitinib in 32 JAK inhibitor-naïve patients (including 47% with HMR mutations), 55% of the patients had an anemia response at any time during treatment [151,152]. SVR35 was noted in all subgroups at week 24, and *JAK2* V617F VAF decreased by >20% in a large proportion of the patients at weeks 12 or 24 compared to baseline regardless of HMR mutation status; however, 31% of the patients experienced grade 3 or 4 thrombocytopenia, an on-target effect of navitoclax [151,152].

Imetelstat is a first-class telomerase inhibitor that is currently being evaluated in the registrational phase 3 trial (IMpactMF; NCT04576156) with the primary endpoint being OS (unprecedented for registrational MF trials) in second-line patients with MF, i.e., those who have failed therapy with a JAK inhibitor [153]. In the phase 2 trial IMbark (NCT02426086), patients in the Arm that received the higher dose of imetelstat (9.4 mg/kg IV every 3 weeks) had prolonged median OS (29.9 months); “triple-negative” patients, who typically have a poor prognosis [25,52], had longer OS compared to the non-“triple-negative” cohort [154]. Furthermore, 25% of the transfusion-dependent patients became transfusion-independent. Additionally, there was a respective ≥25% decrease in the VAFs of the three driver mutations (*JAK2* V617F, *CALR, MPL*) and ≥1 grade decrease in bone marrow fibrosis in 42% and 40.5% of the patients who received 9.4 mg/kg of imetelstat; the latter improvements were correlated with longer OS [154].

## 7. Conclusions

We are in an exciting and dynamic era in MF treatment in light of multiple approved or emerging JAK inhibitors to treat patients presenting with the myeloproliferative and myelodepletive phenotypes [155,156] and the explosive growth of novel agents in advanced clinical development [117,118]. The wide range of new treatments will allow tailored regimens based on the heterogeneous clinical manifestations and molecular markers encountered in the myeloproliferative and myelodepletive phenotypes. The distinct prognostication models applied in PMF and secondary MF (taking into account different clinical features and biology), along with the results of two recent studies that demonstrated the differential prognostic impact of *ASXL1* [29] and *SF3B1* [33] mutations on OS in primary versus secondary MF, further underscore the complex biology underlying the two phenotypes and the necessity of tailored treatments for the two phenotypes.

In the near future, physicians will have the opportunity to design personalized treatments according to each patient’s clinical and genetic profiles, thereby maximizing clinical benefits and improving disease course and outcomes. Beyond simply targeting the JAK-STAT pathway, the type and degree of cytopenias are already being taken into account in selecting from multiple JAK inhibitor options, and the availability of momelotinib will only expand the choices. Although momelotinib and pacritinib are both reasonable options to treat patients presenting with the myelodepletive phenotype (both disease- and treatment-related cytopenias), momelotinib may be the treatment of choice for transfusion-dependent patients who also have progressive splenomegaly and/or constitutional symptoms, whereas pacritinib may be favored when thrombocytopenia is prominent. Navtemadlin or imetelstat may be suitable for the treatment of high-risk MF patients who are resistant to ruxolitinib and have relatively robust blood counts, with imetelstat carrying the promise of significant prolongation of OS post-ruxolitinib. Synergistic combination regimens, such as pelabresib or navitoclax with ruxolitinib, may increase the depth and duration of spleen and symptom responses and improve other aspects of the disease, such as driver mutation burden and bone marrow fibrosis in MF patients presenting with the myeloproliferative phenotype [79,87]. Prolongation of survival, currently the primary endpoint of the pivotal trial of imetelstat, would be a welcome advancement in the field preceded by the transformative impact of ruxolitinib [157,158,159,160,161]. Achievement of RBC transfusion independence is the primary endpoint of the INDEPENDENCE trial of luspatercept and is likely to be seen more and more in registrational trials [87], given that anemia is a cardinal feature of MF and achievement of RBC transfusion independence was correlated with superior OS in the SIMPLIFY-1 trial [162]. The expanding therapeutic landscape in MF engenders optimism and, hopefully, will usher in an era of superior quality of life, improved overall outcomes and prolongation of survival, especially for cytopenic patients who have historically had limited treatment options and a poor prognosis.

## Figures and Tables

**Table 1:** Features encountered in the myeloproliferative and myelodepletive phenotypes of myelofibrosis.

Clinical Features	Myeloproliferative Phenotype	Myelodepletive Phenotype
MF subtype (not exclusive)	More secondary MF	Usually primary MF
Peripheral blood cell counts	Normal or mildly elevated	≥2 cytopenias
RBC counts, hemoglobin	Mild or no anemia	Prominent anemia
Platelet counts	Normal or high	Moderate (50–100 × 10^9^/L) or severe (< 50 × 10^9^/L) thrombocytopenia
WBC counts	Leukocytosis	Leukopenia
RBC transfusion dependence	Usually independent or minimal	More likely to be dependent
Spleen volume	Larger	Smaller
Constitutional symptoms	Abdominal pain, night sweats	Fatigue
Bone marrow fibrosis grade	<2	≥2
Bone marrow cellularity	Usually hypercellular	More likely to be hypocellular
*JAK2* V617F VAF	Higher median (≥50%)	Lower median (<25%)
HMR mutations * (epigenetic or mRNA splicing)	0–1	Multiple
Blast counts	Fewer blasts	Higher blasts
Median overall survival	Longer	Shorter
Risk of leukemic transformation	Lower	Higher
Response to ruxolitinib	High	Limited

* High molecular risk (HMR) mutations: *ASXL1*, *EZH2*, *IDH1*, *IDH2*, *SRSF2*, *U2AF1* Q157. Abbreviations. MF: myelofibrosis; RBC: red blood cell; VAF: variant allele frequency; WBC: white blood cell.
